# Risk of uncomplicated peptic ulcer disease in a cohort of new users of low-dose acetylsalicylic acid for secondary prevention of cardiovascular events

**DOI:** 10.1186/s12876-014-0205-y

**Published:** 2014-12-10

**Authors:** Ana Ruigómez, Saga Johansson, Péter Nagy, Mar Martín-Pérez, Luis A García Rodríguez

**Affiliations:** Spanish Centre for Pharmacoepidemiologic Research (CEIFE), Almirante 28-2, E 28004 Madrid, Spain; AstraZeneca R&D, Pepparedsleden 1, Mölndal, SE-431 83 Sweden

**Keywords:** Acetylsalicylic acid, Nested case–control study, Uncomplicated peptic ulcer disease

## Abstract

**Background:**

The aim of this study was to analyse the risk of uncomplicated peptic ulcer disease (PUD) in a cohort of new users of low-dose acetylsalicylic acid (ASA) for secondary prevention of cardiovascular events in a UK primary care setting.

**Methods:**

New users of low-dose ASA for secondary prevention of cardiovascular events, aged 50-84 years in 2000-2007, were identified from The Health Improvement Network. Among those 38,975 individuals, 309 patients were considered to be incident cases of uncomplicated PUD. Incidence of uncomplicated PUD was calculated and a nested case–control analysis adjusted for potential confounding factors was performed to calculate the odds ratios (ORs) for the association of potential risk factors with uncomplicated PUD.

**Results:**

The crude incidence of uncomplicated PUD was 1.41 per 1000 person-years (95% confidence interval [CI], 1.26-1.58). Individuals with a history of PUD were more likely to develop uncomplicated PUD than those without such a history (hazard ratio [HR], 2.22, 95% CI, 1.60-3.09). In nested case–control analyses, the risk of uncomplicated PUD was associated with current use of non-steroidal anti-inflammatory drugs, oral steroids or acid suppressants. Other risk factors for developing uncomplicated PUD included smoking, stress, depression, anaemia and social deprivation.

**Conclusion:**

Our results indicate that several risk factors significantly increase the risk of development of uncomplicated PUD in new users of low-dose ASA. Therefore, physicians should monitor ASA users for gastrointestinal symptoms and signs of ulcer, particularly if they have additional risk factors.

**Electronic supplementary material:**

The online version of this article (doi:10.1186/s12876-014-0205-y) contains supplementary material, which is available to authorized users.

## Background

Current evidence-based guidelines recommend the long-term use of acetylsalicylic acid (ASA) for the secondary prevention of cardiovascular events [1,2]. Use of ASA has, however, been shown to increase the risk of gastrointestinal complications, including upper gastrointestinal bleeding [3] and uncomplicated peptic ulcer disease (PUD) [4]. Upper gastrointestinal symptoms have been shown to affect patients’ health-related quality of life [5] and may lead to discontinuation of ASA therapy [6-9], leaving patients at elevated risk of cardiovascular events [10-12]. Moreover, uncomplicated PUD symptoms may influence the prescribing behaviour of primary care physicians (PCPs). It is therefore important to understand the additional factors associated with an increased risk of PUD in patients using ASA for the secondary prevention of cardiovascular events.

Although these additional risk factors have been studied for complicated PUD [3], data remain scarce for uncomplicated PUD. Uncomplicated PUD contributes to the overall health burden of PUD and may lead to complications in some patients [13,14]; even in the absence of overt bleeding, uncomplicated PUD may cause anaemia [15]. In a previous observational study in the UK in 1997-2005, we showed that the overall incidence of uncomplicated PUD was 0.75 cases per 1000 person-years in the general UK population [16]. A similar incidence of uncomplicated PUD was also reported in a population-based study in Denmark [13].

The aims of the present study were to estimate the incidence of symptomatic uncomplicated PUD among a cohort of individuals starting low-dose ASA therapy for the secondary prevention of cardiovascular events in a UK primary care setting and to determine the main factors associated with an increased risk of uncomplicated PUD in a nested case–control analysis.

## Methods

### Data source

A retrospective cohort study was performed using data from The Health Improvement Network (THIN), a computerized primary care database containing anonymized records for individuals currently registered with participating primary care practices in the UK. THIN is age, sex and geographically representative of the UK population [17] and has been extensively validated for use in epidemiological studies [18]. Data recorded in THIN include patient demographics, details of consultations with PCPs, information about consultant referrals and hospitalizations, laboratory test results, diagnoses and prescriptions. The Read classification is used to code specific diagnoses [19,20], and a drug dictionary based on data from the Gemscript classification is used to record prescriptions [21]. Ethical approval for the collection of data in THIN database was obtained from a Multicentre Research Ethics Committee (NHS; MREC reference number: 08/H0305/49).

### Study cohort

A previously identified cohort of patients who received a first-ever prescription for low-dose ASA (75-300 mg/day) for secondary prevention of cardiovascular ischaemic diseases or cerebrovascular ischaemic events and who were aged 50-84 years between 1 January 2000 and 31 December 2007 was used for the present study [11]. Patients were required to have been registered for at least 2 years with their PCP, and to have at least 1 year of computerized prescription history. The date of their first-ever recorded prescription for low-dose ASA for secondary prevention of cardiovascular events was defined as their start date. All patients who had a prescription for low-dose ASA recorded before their start date were excluded, as were patients with a recorded diagnosis of alcohol abuse or cancer. Individuals aged 70 years or older with a follow-up longer than 1 year and less than two health contacts during their follow-up period were also excluded (proxy for incomplete data recording). The total study cohort consisted of 38,975 individuals, who were followed up to identify incident cases of uncomplicated PUD.

### Uncomplicated PUD case ascertainment

All study cohort members were followed up for a mean of 5.6 years from the first day after their start date to the earliest of the following endpoints: first recorded diagnosis of uncomplicated PUD, cancer, alcohol abuse or alcohol-related disease; reaching the age of 85 years; date of the last practice data collection; death; or end of the study period (30 September 2011).

We identified 555 patients with a first computer-recorded Read code suggesting uncomplicated PUD during follow-up. For all of them, we requested free-text comments close to the date of the Read code (a week before and a month after that date) and comments associated with upper gastrointestinal-related diagnoses recorded any time before and up to 6 months after the computer-detected uncomplicated PUD diagnosis. After removal of personal identifiers and information on drug use, we manually reviewed the profiles of these 555 patients. Individuals were considered to have uncomplicated PUD if a clinical diagnosis had been made during a specialist visit or hospitalization, and if the site of the ulcer was located in the stomach or duodenum without any major complication, bleeding or perforation. Following this review, 327 patients were classified as definite uncomplicated PUD cases, 20 as possible cases (insufficient information for ascertainment) and 208 as non-cases. Among the 327 definite uncomplicated PUD cases, almost 90% of records showed that an endoscopy had been performed. A little over half of definite cases of uncomplicated PUD had a *Helicobacter pylori* test recorded on or near the date of diagnosis (n = 179, 54.7%).

To confirm the validity of our case ascertainment further, we sent a questionnaire to the corresponding PCPs requesting confirmation and copies of paper-based records for 100 patients randomly sampled from the definite cases (n = 96) and possible cases (n = 4). We received records for 98 patients. The uncomplicated PUD diagnosis was confirmed by the PCPs for 76 patients. The confirmation rate among the definite cases was 80% and only 25% among possible cases. We retained as cases all definite cases confirmed by a questionnaire (n = 75) and those definite cases for which we did not have a questionnaire (n = 233). Due to the low confirmation rate among possible cases, we only retained the single patient initially classified as possible, whose diagnosis was confirmed by the PCP. The majority of patients who were not retained as uncomplicated PUD cases had a discharge letter with a diagnosis of a complication (e.g. bleeding) not recorded in their computerized file.

**Figure 1:**
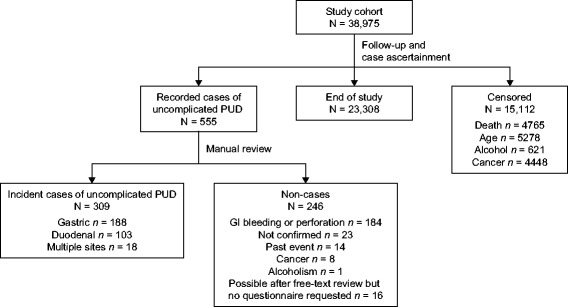
**Study design and case ascertainment.** Abbreviations: *GI-*Gastrointestinal; *PUD*-Peptic ulcer disease.

Following this two-step review process, 309 patients were considered to be incident cases of uncomplicated PUD: 308 individuals from those initially classified as definite and 1 from the possible cases (Figure 1). The index date was defined as the date of the computer-recorded diagnosis (n = 144) or the date of the first symptom leading to the diagnosis of PUD (n = 165), whichever occurred first. When the date of the first symptom was used as the index date, the mean time to the computer-recorded diagnosis of uncomplicated PUD was 35 days. The peptic ulcer was located in the stomach for 188 patients (61%), the duodenum for 103 (33%) and multiple sites (stomach and duodenum) for 18 (6%).

### Selection of controls

A date within the study period was generated at random for each member of the study cohort, excluding PUD cases. If the random date was included in the individual’s person-time contribution, we marked that person as an eligible control. The random date for each control individual was used as the index date in the nested case–control analysis. In total, 2000 controls, frequency-matched to cases by age (within 1 year), sex and follow-up time (interval between start date and index date), were randomly selected from the pool of eligible controls.

### Risk factor ascertainment

Information on patient demographics, baseline characteristics, comorbidities and comedications was collected from THIN. In the cohort analyses, we investigated the following potential risk factors: age, sex, year of start date, ASA indication (myocardial infarction, unstable angina, ischaemic heart disease and cerebrovascular disease), history of PUD and use of proton pump inhibitors (PPIs). A history of PUD any time before the start date was defined as any record of peptic ulcer symptoms, or a diagnosis of uncomplicated or complicated PUD. We defined PPI users at their start date as those who had received at least one prescription for a PPI in the month before or in the week after their first ASA prescription (start date).

For nested case–control analyses, information on comorbidities and other potential risk factors, such as smoking, alcohol use and body mass index (BMI), were collected from patients’ records at any time before the index date. We assessed gastroesophageal reflux disease (GERD) and symptoms related to PUD, including vomiting, nausea, epigastric pain, dyspepsia, heartburn and gastritis between the start date and the index date. In addition, data on the number of PCP visits, referrals and hospitalizations recorded for each patient were collected for the year before their index date. Finally, for the case–control analyses, information on patients’ drug exposure was assessed between their start date and index date, and was categorized as follows: *current use*, when the supply of the most recent prescription lasted until the index date or ended in the 30 days preceding the index date; *past use*, when the supply of the most recent prescription ended 31-365 days before the index date; and *non-use,* when the most recent prescription ended more than 365 days before the index date or there was no recorded use at any time between the start date and the index date.

### Statistical analysis

The overall incidence of uncomplicated PUD and associated 95% confidence interval (CI) was determined along with age- and sex-specific estimates. We also calculated the incidence of uncomplicated PUD in subgroups of ASA users with and without a history of PUD before their start date. The incidence of uncomplicated PUD in new users of low-dose ASA who were exposed to a PPI at their start date was also compared with the incidence in those who were not exposed to a PPI at their start date. Nelson–Aalen cumulative hazard estimates were calculated for ASA users with and without a history of PUD and compared using a log-rank test. Hazard ratios (HRs) and associated 95% CIs were calculated using Cox regression analyses adjusted for age, sex, year of start date, ASA indication, PPI use and history of PUD. All variables were ascertained at the start date.

Nested case–control analyses were performed to estimate the contribution of various risk factors to the development of uncomplicated PUD during follow-up. Odds ratios (ORs) and associated 95% CIs were calculated by unconditional multiple logistic regression models. All estimates were adjusted for frequency-matched variables (age, sex, follow-up time) and for health service utilization (PCP visits and referrals), smoking, and use of acid-suppressing drugs, non-steroidal anti-inflammatory drugs (NSAIDs), ASA and paracetamol.

Statistical analyses were performed using Stata® version 12.0 (StataCorp LP, College Station, TX, USA).

## Results

### Incidence of uncomplicated PUD

**Figure 2:**
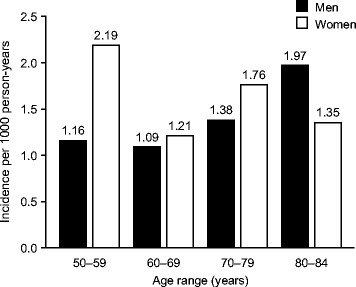
**Incidence of uncomplicated peptic ulcer disease by age and sex.**

**Figure 3:**
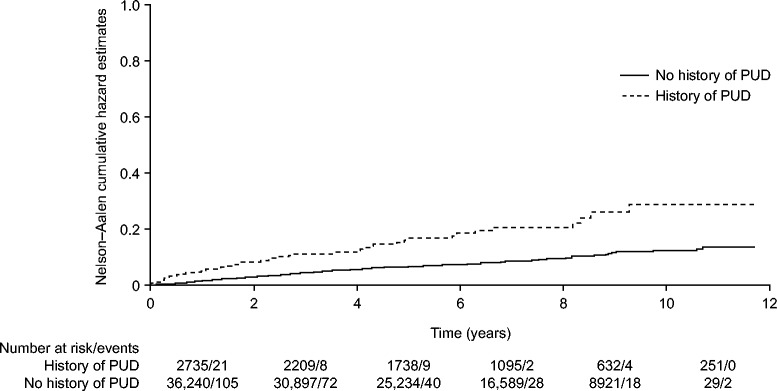
**Nelson–Aalen cumulative hazard estimates for the development of uncomplicated peptic ulcer disease.** Abbreviation: *PUD*-Peptic ulcer disease.

The crude incidence of uncomplicated PUD in new users of low-dose ASA was 1.41 per 1000 person-years (95% CI, 1.26-1.58). For women and men, the incidences were 1.56 per 1000 person-years (95% CI, 1.33-1.83) and 1.30 per 1000 person-years (95% CI, 1.11-1.52), respectively. When stratified by age, women aged 50-59 years had a higher incidence of uncomplicated PUD (2.19 per 1000 person-years, 95% CI, 1.43-3.34) than men in the corresponding age group (1.16 per 1000 person-years, 95% CI, 0.77-1.76). For patients aged 80-84, the opposite was observed (Figure 2). A higher incidence of uncomplicated PUD was observed in new users of ASA with a history of PUD (3.03 per 1000 person-years, 95% CI, 2.26-4.08) than in those without such a history (1.30 per 1000 person-years, 95% CI, 1.15-1.46). In a Nelson–Aalen cumulative hazards analysis, patients with a history of PUD had a significantly greater probability of developing uncomplicated PUD than patients without such a history (log-rank test, p < 0.001; Figure 3). New users of ASA exposed to a PPI at their start date had a higher incidence of uncomplicated PUD (1.84 per 1000 person-years, 95% CI, 1.45-2.32) than those not exposed (1.32 per 1000 person-years, 95% CI, 1.16-1.50).

### Risk factors for uncomplicated PUD

In an adjusted Cox regression analysis, sex, year of start date, ASA indication and use of PPIs did not appear to be associated with an increased risk of uncomplicated PUD (Table 1). Older patients (aged 80-84 years) were more likely to develop uncomplicated PUD than patients aged 50-59 years (adjusted HR, 1.69; 95% CI, 1.05-2.74). New users of low-dose ASA with a history of PUD were about twofold more likely to develop uncomplicated PUD than those without such a history (adjusted HR, 2.22; 95% CI, 1.60-3.09).

**Table 1:** **Hazard ratios for uncomplicated peptic ulcer disease associated with factors assessed at the start date (cohort analysis)**

	**Adjusted HR (95% CI)** ^**a**^
Sex	
Male	1 (-)
Female	1.19 (0.95-1.50)
Age at start date, years	
50-59	1 (-)
60-69	1.08 (0.79-1.47)
70-79	1.26 (0.92-1.73)
80-84	1.69 (1.05-2.74)
Year of start date	
2000-2001	1 (-)
2002-2003	1.02 (0.77-1.35)
2004-2005	0.84 (0.61-1.17)
2006-2007	0.84 (0.57-1.22)
ASA indication	
Cerebrovascular disease	1 (-)
Myocardial infarction	1.12 (0.82-1.53)
Ischaemic heart disease	1.06 (0.81-1.39)
Unstable angina	1.25 (0.60-2.59)
PPI use at start date	
No	1 (-)
Yes	1.23 (0.93-1.62)
History of PUD at start date	
No	1 (-)
Yes	2.22 (1.60-3.09)

^**a**^HRs estimated by Cox regression analyses and adjusted for all variables in the table.

*Abbreviations:* *ASA*-Acetylsalicylic acid; *CI*-Confidence interval; *HRs*-Hazard ratios; *PPI*-Proton pump inhibitor; *PUD*-Peptic ulcer disease.

In nested case–control analyses, smoking was a predictor of uncomplicated PUD development (adjusted OR, 1.96; 95% CI, 1.37-2.80) (Table 2). We further examined the association between smoking and uncomplicated PUD according to the ulcer site (stomach or duodenum). Smokers had a threefold greater risk of duodenal ulcer than non-smokers (adjusted OR, 3.40; 95% CI: 1.94-5.97), whereas the risk of gastric ulcer was increased by less than twofold (adjusted OR, 1.64; 95% CI, 1.04-2.59). The risk of developing uncomplicated PUD was twofold greater for patients with four or more referrals to a specialist during the year preceding their index date than for those who had not been referred (adjusted OR, 1.98; 95% CI, 1.30-3.00). Individuals living in urban areas were more likely to experience uncomplicated PUD than those living in rural areas (adjusted OR, 3.21; 95% CI, 1.46-7.04). Patients living in the most deprived areas (as measured by the Townsend deprivation index) had an increased risk of uncomplicated PUD compared with those living in the least deprived areas (adjusted OR, 1.56; 95% CI, 1.01-2.39). Alcohol use and BMI did not have a statistically significant effect on the risk of uncomplicated PUD. Among the comorbidities investigated, an increased risk of uncomplicated PUD in new users of low-dose ASA was associated with anaemia (adjusted OR, 2.53; 95% CI, 1.82-3.53), stress (adjusted OR, 1.58; 95% CI, 1.06-2.33) and depression (adjusted OR, 1.38; 95% CI, 1.04-1.83). The presence of uncomplicated PUD symptoms (e.g. nausea, vomiting and epigastric pain) after initiation of ASA treatment was a predictor of uncomplicated PUD (adjusted OR, 2.09; 95% CI, 1.56-2.81), whereas a diagnosis of GERD during follow-up was not (adjusted OR, 1.13; 95% CI, 0.81-1.57).

**Table 2:** **Odds ratios for the risk of uncomplicated peptic ulcer disease associated with patient characteristics and comorbidities (nested case–control analysis)**

	**Controls N = 2000** ***n*** **(%)**	**Uncomplicated PUD cases N = 309** ***n*** **(%)**	**Adjusted OR (95% CI)** ^**a**^
Sex			
Male	1046 (52.3)	161 (52.1)	NA
Female	954 (47.7)	148 (47.9)	NA
Age at index date, years			
50-59	250 (12.5)	43 (13.9)	NA
60-69	575 (28.7)	86 (27.8)	NA
70-79	859 (43.0)	129 (41.7)	NA
80-84	316 (15.8)	51 (16.5)	NA
Follow-up time, months			
< 6	211 (10.5)	38 (12.3)	NA
6-12	227 (11.4)	33 (10.7)	NA
13-24	335 (16.8)	55 (17.8)	NA
25-48	538 (26.9)	80 (25.9)	NA
> 48	689 (34.5)	103 (33.3)	NA
Visits to PCP^b^			
< 3	62 (3.1)	3 (1.0)	1 (-)
4-9	544 (27.2)	47 (15.2)	1.35 (0.40-4.52)
≥ 10	1394 (69.7)	259 (83.8)	2.03 (0.61-6.76)
Referrals^b^			
0	466 (23.3)	37 (12.0)	1 (-)
1-3	834 (41.7)	118 (38.2)	1.46 (0.97-2.20)
≥ 4	700 (35.0)	154 (49.8)	1.98 (1.30-3.00)
Hospitalizations^b^			
0	1584 (79.2)	213 (68.9)	1 (-)
≥ 1	416 (20.8)	96 (31.1)	1.21 (0.90-1.63)
Smoking			
Non-smoker	800 (40.0)	105 (34.0)	1 (-)
Smoker	267 (13.4)	67 (21.7)	1.96 (1.37-2.80)
Ex-smoker	892 (44.6)	135 (43.7)	1.01 (0.76-1.34)
Unknown	41 (2.1)	2 (0.6)	0.48 (0.11-2.08)
BMI, kg/m^2^			
11-19	67 (3.4)	15 (4.9)	1.11 (0.59-2.11)
20-24	489 (24.4)	86 (27.8)	1 (-)
25-29	781 (39.1)	114 (36.9)	0.81 (0.59-1.11)
≥ 30	510 (25.5)	80 (25.9)	0.83 (0.59-1.18)
Unknown	153 (7.6)	14 (4.5)	0.69 (0.37-1.31)
Alcohol use, units per week			
0	88 (4.4)	19 (6.1)	1 (-)
1-4	377 (18.9)	58 (18.8)	0.67 (0.37-1.21)
5-15	395 (19.8)	56 (18.1)	0.64 (0.35-1.17)
≥ 16	148 (7.4)	22 (7.1)	0.65 (0.31-1.32)
Unknown	992 (49.6)	154 (49.8)	0.66 (0.38-1.16)
Practice location			
Rural	235 (6.8)	7 (2.3)	1 (-)
Urban	1382 (69.1)	233 (75.4)	3.21 (1.46-7.04)
Town	240 (12.0)	28 (9.1)	2.38 (1.00-5.68)
Unknown	243 (12.2)	41 (13.3)	2.75 (1.18-6.42)
Townsend deprivation index			
1 (least deprived)	489 (24.4)	59 (19.1)	1 (-)
2	435 (21.8)	55 (17.8)	1.09 (0.73-1.63)
3	411 (20.5)	68 (22.0)	1.29 (0.88-1.90)
4	382 (19.1)	68 (22.0)	1.35 (0.91-1.99)
5 (most deprived)	54 (17.5)	54 (17.5)	1.56 (1.01-2.39)
Unknown	5 (1.6)	5 (1.6)	0.52 (0.20-1.37)
Comorbidities			
Cerebrovascular disease^c^	629 (31.5)	91 (29.4)	0.89 (0.68-1.17)
Ischaemic heart disease^c^	1382 (69.1)	221 (71.5)	1.07 (0.81-1.41)
Myocardial infarction^c^	514 (25.7)	97 (31.4)	1.26 (0.96-1.66)
Hypertension^c^	1223 (61.2)	180 (58.3)	0.82 (0.63-1.06)
Hyperlipidaemia^c^	697 (34.9)	114 (36.9)	1.08 (0.83-1.41)
Diabetes mellitus^c^	328 (16.4)	60 (19.4)	1.08 (0.78-1.50)
Gout^c^	176 (8.8)	22 (7.1)	0.70 (0.43-1.14)
Rheumatoid arthritis^c^	55 (2.8)	20 (6.5)	1.46 (0.83-2.58)
Osteoarthritis^c^	858 (42.9)	157 (50.8)	1.13 (0.87-1.46)
COPD^c^	158 (7.9)	34 (11.0)	1.08 (0.71-1.65)
Asthma^c^	324 (16.2)	54 (17.5)	0.82 (0.59-1.15)
Anaemia^c^	177 (8.8)	70 (22.7)	2.53 (1.82-3.53)
Stress^c^	158 (7.9)	42 (13.6)	1.58 (1.06-2.33)
Anxiety^c^	351 (17.6)	70 (22.7)	1.21 (0.89-1.64)
Depression^c^	474 (23.7)	103 (33.3)	1.38 (1.04-1.83)
IBS^c^	129 (6.5)	25 (8.1)	1.06 (0.66-1.71)
GERD^c^	280 (14.0)	60 (19.4)	1.13 (0.81-1.57)
Uncomplicated PUD symptoms^d^	471 (23.6)	133 (43.0)	2.09 (1.56-2.81)

^a^OR adjusted for age, sex, follow-up time, health service utilization (PCP visits and referrals), smoking and drug use during the study period (gastroprotective drugs, NSAIDs, ASA and paracetamol).

^b^In year before the index date.

^c^Diagnosed before the index date. Relative to being free from the comorbidity.

^d^Diagnosed between the start date and the index date. Relative to being free from symptoms.

*Abbreviations:* *ASA*-Acetylsalicylic acid; *BMI*-Body mass index; *CI*-Confidence interval; *COPD*-Chronic obstructive pulmonary disease; *GERD*-Gastroesophageal reflux disease; *IBS*-Irritable bowel syndrome; *NA*-Not assessed; *NSAIDs*-Non-steroidal anti-inflammatory drugs; *OR*-Odds ratio; *PCP*-Primary care physician; *PUD*-Peptic ulcer disease.

Overall, current use of NSAIDs (selective cyclooxygenase-2 [COX-2] inhibitors and traditional NSAIDs) was associated with a significantly increased risk of uncomplicated PUD (adjusted OR, 1.50; 95% CI, 1.06-2.13). When considered separately, the association was significant for COX-2 inhibitors (adjusted OR, 2.33; 95% CI, 1.13-4.79) but was not for traditional NSAIDs (adjusted OR, 1.36; 95% CI, 0.93-1.99). Current use of oral steroids and current use of paracetamol were also associated with an increased risk of PUD (adjusted OR, 1.88; 95% CI, 1.09-3.25 and adjusted OR, 1.45; 95% CI, 1.08-1.95, respectively) (Table 3). An association between acid-suppressing drugs and uncomplicated PUD was observed: current users of PPIs and current users of histamine-2 receptor antagonists (H_2_RAs) were significantly more likely to have uncomplicated PUD than non-users of these drugs (adjusted OR, 1.68; 95% CI, 1.26-2.25 and adjusted OR, 2.26; 95% CI, 1.34-3.82, respectively). When current users of PPI therapy were divided into subgroups, current use of PPI therapy initiated more than 30 days after the start date was significantly associated with an increased risk of uncomplicated PUD (adjusted OR, 2.38; 95% CI, 1.65-3.42), whereas current use of PPI intitiated before the start date or within the 30 days after the start date was not significantly associated (adjusted OR, 1.25; 95% CI, 0.86-1.80).

**Table 3:** **Odds ratios for the risk of uncomplicated peptic ulcer disease associated with current use of medications (nested case–control analysis)**

	**Controls N = 2000** ***n*** **(%)**	**Uncomplicated PUD cases N = 309** ***n*** **(%)**	**Adjusted OR (95% CI)** ^**a**^
NSAIDs	206 (10.3)	57 (18.4)	1.50 (1.06-2.13)
Selective COX-2 inhibitors	28 (1.4)	13 (4.2)	2.33 (1.13-4.79)
tNSAIDs	178 (8.9)	44 (14.2)	1.36 (0.93-1.99)
Paracetamol	492 (24.6)	114 (36.9)	1.45 (1.08-1.95)
Low-dose ASA	1515 (75.8)	245 (79.3)	1.33 (0.87-2.04)
Clopidogrel	157 (7.9)	29 (9.4)	0.94 (0.60-1.48)
Oral anticoagulants	84 (4.2)	8 (2.6)	0.68 (0.31-1.48)
Dipyridamole	62 (3.1)	9 (2.9)	0.78 (0.37-1.62)
PPIs	453 (22.7)	107 (34.6)	1.68 (1.26-2.25)
Used on start date^b^	266 (13.3)	48 (15.5)	1.25 (0.86-1.80)
Use initiated after start date^c^	187 (9.3)	59 (19.1)	2.38 (1.65-3.42)
H_2_RAs	60 (3.0)	23 (7.4)	2.26 (1.34-3.82)
Oral steroids	58 (2.9)	21 (6.8)	1.88 (1.09-3.25)
SSRIs	103 (5.2)	27 (8.7)	1.45 (0.91-2.32)
Tricyclic antidepressants	105 (5.3)	19 (6.1)	1.03 (0.61-1.74)
Statins	1431 (71.6)	220 (71.2)	0.89 (0.65-1.23)

^a^OR adjusted for age, sex, follow-up time, health service utilization (PCP visits and referrals), smoking and drug use during study period (gastroprotective drugs, NSAIDs, ASA and paracetamol). Relative to non-use of drug.

^b^PPI therapy in use on the start date or started within the 30 days after the start date.

^c^PPI therapy started after the first 30 days of follow-up.

*Abbreviations:* *ASA-*Acetylsalicylic acid; *CI*-Confidence interval; *COX-2*-Cyclooxygenase-2; *H*_*2*_ *RA*s-Histamine-2 receptor antagonists; *NSAIDs*-Non-steroidal anti-inflammatory drugs; *OR*-Odds ratio; *PCP*-Primary care physician; *PPIs*-Proton pump inhibitors; *PUD*-Peptic ulcer disease; *SSRIs*-Selective serotonin reuptake inhibitors; *tNSAIDs*-Traditional non-steroidal anti-inflammatory drugs.

In a secondary analysis, regression models were computed only for current users of low-dose ASA at the index date. Estimates of risks for the development of uncomplicated PUD in current low-dose ASA users were comparable to those observed for the whole cohort for all variables (Additional file 1: Table S1 and Additional file 2: Table S2).

## Discussion

Few epidemiological studies have investigated risk factors for uncomplicated PUD separately from risk factors for complicated PUD, particularly in a population of new users of ASA. The overall incidence of uncomplicated PUD in this population was 1.41 per 1000 person-years and was higher than the incidence calculated for the general population [13,16]. A similar incidence of upper gastrointestinal bleeding was observed in the same cohort of new users of ASA (1.12 per 1000 person-years) [3].

Our results indicate that the development of uncomplicated PUD in new users of low-dose ASA for the prevention of cardiovascular events was associated with social deprivation, a history of PUD, smoking, stress, depression, anaemia and use of NSAIDs, COX-2 inhibitors and oral steroids. This was in line with the risk factors for uncomplicated PUD observed in the general population [4,13,22]. Many of the risk factors for uncomplicated PUD identified in this study have also been demonstrated to increase the risk of complications of PUD in the general population; previous studies have reported an increased risk of upper gastrointestinal bleeding associated with smoking [23-27], use of NSAIDs [24,25,27-29], use of COX-2 inhibitors [24] and use of high doses of oral steroids [24].

Among the risk factors identified in our study, a history of PUD has been shown by other investigators to increase the risk of upper gastrointestinal bleeding in ASA users [3]. The use of drugs such as NSAIDs, COX-2 inhibitors and oral steroids has also been shown to increase the risk of upper gastrointestinal bleeding in users of ASA [3,24]. In a similar population of new ASA users, patients using NSAIDs were almost three times more likely to develop upper gastrointestinal bleeding than non-users of NSAIDs [3]. This is higher than the observed risk for uncomplicated PUD in the current study, and may suggest that some cases of PUD remain undiagnosed until complications occur.

Current use of acid-suppressing therapy (PPIs or H_2_RAs) was associated with an increased risk of developing uncomplicated PUD in new ASA users. A similar association between the use of PPIs and an increased risk of PUD recurrence was observed in Finland [30]. In our study, the association between PPIs and uncomplicated PUD was mainly observed for a subgroup of individuals whose PPI therapy started more than 30 days after their first ASA prescription. In contrast, no increased risk was observed for individuals whose PPI therapy had been started before or at the same time as their first ASA prescription. In line with observations from others [31], this association is likely to be explained by confounding by indication. PPIs prescribed at some point after initiating ASA therapy might be a marker of developing upper gastrointestinal symptoms during ASA therapy in patients who may be at greater background ulcer risk than individuals not starting PPI therapy during their follow-up. Similarly, the increased risk of uncomplicated PUD observed in current users of H_2_RAs is likely due to confounding by indication, whereby H_2_RAs are prescribed to treat the symptoms of uncomplicated PUD.

The increased risk of uncomplicated PUD associated with the use of paracetamol suggests that paracetamol may potentiate the ulcerogenicity of ASA. This association, however, remains unclear and may be spurious; patients with diagnosed uncomplicated PUD may be preferentially prescribed paracetamol as a safer alternative to NSAIDs.

Among the comorbidities investigated, anaemia was associated with the highest risk of developing uncomplicated PUD. In the absence of overt bleeding, this might be explained by undiagnosed gastrointestinal microbleeding caused by uncomplicated PUD [15]. The causal direction of this association, however, remains unclear and the hypothesis that anaemia might be a predictor of uncomplicated PUD cannot be ruled out.

The association between *Helicobacter pylori* infection and PUD is well established [26,32,33]. In our study, the incomplete recording of *H. pylori* infection in definite cases together with the overall lack of *H. pylori* status in the general population, including controls, prevented us from analysing the role of *H. pylori*.

The present study has the strength of using a large primary care database that is representative of the UK population and has been validated for use in epidemiological studies [17,18]. Additionally, THIN is likely to afford more accurate estimates of uncomplicated PUD incidence than hospital-based databases [34]. It should be noted that THIN does not report use of over-the-counter (OTC) medications. Prescription medications, however, are free for patients aged 60 years or older in the UK, and health care is easily accessed, which is likely to encourage prescription rather than OTC medication use. In our study, most patients (87.3%) were aged 60 years or older and therefore misclassification of drug use due to medications being obtained OTC should not greatly affect our study results.

Another key strength of the study is that potential cases of uncomplicated PUD were carefully ascertained by manually reviewing patients’ records, including free-text comments. Despite this careful process, however, when PCPs were contacted to confirm the diagnosis of uncomplicated PUD, the confirmation rate was about 80%. Most of the misclassification was due to cases of complicated PUD. It should also be noted that this study only reflects symptomatic uncomplicated PUD; in the absence of endoscopy, asymptomatic uncomplicated PUD remains undiagnosed.

## Conclusions

In conclusion, every year, uncomplicated PUD develops in approximately 1-2 patients per 1000 taking low-dose ASA for the secondary prevention of cardiovascular events, adding to the burden of disease associated with complicated PUD in this population. This incidence rises to 3 patients per 1000 in those with a history of PUD. Other factors that significantly increase the risk of uncomplicated PUD in new ASA users include smoking, depression, anaemia, current use of acid-suppressing drugs and NSAIDs. Therefore, physicians should closely monitor ASA users for gastrointestinal symptoms and signs of ulcers, particularly if they have additional risk factors. This may allow early diagnosis of uncomplicated PUD and help to reduce the development of complications.

### Consent

We used The Health Improvement Network (THIN) primary care data for this study. The company that owns THIN (Cegedim Strategic Data Medical Research) has received ethical approval from the South East Research Ethics Committee (REC) to supply anonymized, pre-collected primary care data for scientific research. Patients can opt out of having their depersonalized records collected. Therefore, patient consent is not required when working with anonymized records from THIN database.

## Additional files

Additional file 1: Table S1. Odds ratios for the risk of uncomplicated peptic ulcer disease associated with patient characteristics and comorbidities, nested case–control analysis restricted to current users of low-dose ASA. Additional file 2: Table S2. Odds ratios for the risk of uncomplicated peptic ulcer disease associated with current use of medications, nested case–control analysis restricted to current users of low-dose ASA at their index date.

## Abbreviations

ASA: Acetylsalicylic acid
BMI: Body mass index
CI: Confidence interval
COX-2: Cyclooxygenase-2
GERD: Gastroesophageal reflux disease
H_2_RA: Histamine-2 receptor antagonist
HR: Hazard ratio
NSAID: Non-steroidal anti-inflammatory drug
OR: Odds ratio
OTC: Over-the-counter
PCP: Primary care physician
PPI: Proton pump inhibitor
PUD: Peptic ulcer disease
THIN: The Health Improvement Network
