# Supplementary material for: Risk of uncomplicated peptic ulcer disease in a cohort of new users of low-dose acetylsalicylic acid for secondary prevention of cardiovascular events
Source: BMC Gastroenterol. 2014 Dec 10;14:205. doi: 10.1186/s12876-014-0205-y (PMC4272555; doi:10.1186/s12876-014-0205-y)
Supplement: Additional file 1: Table S1. — Odds ratios for the risk of uncomplicated peptic ulcer disease associated with patient characteristics and comorbidities, nested case–control analysis restricted to current users of low-dose ASA. [file 12876_2014_205_MOESM1_ESM.doc]

**Supplementary Table 1 Odds ratios for the risk of uncomplicated peptic ulcer disease associated with patient characteristics and comorbidities, nested case–control analysis restricted to current users of low-dose ASA**

|  | **Controls**  **N = 1515**  ***n* (%)** | **Uncomplicated PUD cases**  **N = 245**  ***n* (%)** | **Adjusted**  **OR (95% CI)^a^** |
| --- | --- | --- | --- |
| Sex | | | |
| Male | 817 (53.9) | 128 (52.2) | NA |
| Female | 698 (46.1) | 117 (47.8) | NA |
| Age at index date, years | | | |
| 50-59 | 167 (11.0) | 32 (13.1) | NA |
| 60-69 | 427 (28.2) | 67 (27.3) | NA |
| 70-79 | 675 (44.6) | 103 (42.0) | NA |
| 80-84 | 246 (16.2) | 43 (17.6) | NA |
| Follow-up time, months | | | |
| < 6 | 192 (12.7) | 32 (13.1) | NA |
| 6-12 | 173 (11.4) | 29 (11.8) | NA |
| 13-24 | 269 (17.8) | 44 (18.0) | NA |
| 25-48 | 382 (25.2) | 68 (27.8) | NA |
| > 48 | 499 (32.9) | 72 (29.4) | NA |
| Visits to PCP^b^ | | | |
| < 3 | 41 (2.7) | 3 (1.2) | 1 (-) |
| 4-9 | 420 (27.7) | 37 (15.1) | 0.93 (0.27-3.22) |
| ≥ 10 | 1054 (69.6) | 205 (83.7) | 1.45 (0.42-4.96) |
| Referrals^b^ | | | |
| 0 | 363 (24.0) | 31 (12.7) | 1 (-) |
| 1-3 | 644 (42.5) | 96 (39.2) | 1.49 (0.95-2.35) |
| ≥ 4 | 508 (33.5) | 118 (48.2) | 1.99 (1.25-3.18) |
| Hospitalizations^b^ | | | |
| 0 | 1203 (79.4) | 169 (69.0) | 1 (-) |
| ≥ 1 | 312 (20.6) | 76 (31.0) | 1.27 (0.90-1.79) |
|  | | | |
| Smoking | | | |
| Non‑smoker | 604 (39.9) | 79 (32.2) | 1 (-) |
| Smoker | 196 (12.9) | 57 (23.3) | 2.35 (1.56-3.52) |
| Ex‑smoker | 683 (45.1) | 107 (43.7) | 1.06 (0.76-1.48) |
| Unknown | 32 (2.1) | 2 (0.8) | 0.76 (0.17-3.33) |
| BMI, kg/m^2^ | | | |
| 11-19 | 53 (3.5) | 12 (4.9) | 1.03 (0.50-2.13) |
| 20-24 | 368 (24.3) | 67 (27.3) | 1 (-) |
| 25-29 | 595 (39.3) | 91 (37.1) | 0.81 (0.56-1.16) |
| ≥ 30 | 382 (25.2) | 62 (25.3) | 0.83 (0.56-1.25) |
| Unknown | 117 (7.7) | 13 (5.3) | 0.81 (0.41-1.61) |
| Alcohol use, units per week | | | |
| 0 | 67 (4.4) | 18 (7.3) | 1 (-) |
| 1-4 | 293 (19.3) | 44 (18.0) | 0.52 (0.27-0.99) |
| 5-15 | 295 (19.5) | 47 (19.2) | 0.58 (0.31-1.12) |
| ≥ 16 | 103 (6.8) | 18 (7.3) | 0.61 (0.28-1.35) |
| Unknown | 757 (50.0) | 118 (48.2) | 0.54 (0.30-0.98) |
| Practice location | | | |
| Rural | 98 (6.5) | 5 (2.0) | 1 (-) |
| Urban | 1035 (68.3) | 185 (75.5) | 3.41 (1.34-8.63) |
| Town | 185 (12.2) | 23 (9.4) | 2.45 (0.89-6.79) |
| Unknown | 197 (13.0) | 32 (13.1) | 2.58 (0.95-7.01) |
| Townsend deprivation index | | | |
| 1 (least deprived) | 370 (24.4) | 45 (18.4) | 1 (-) |
| 2 | 316 (20.9) | 47 (19.2) | 1.26 (0.80-1.98) |
| 3 | 321 (21.2) | 54 (22.0) | 1.30 (0.84-2.03) |
| 4 | 288 (19.0) | 49 (20.0) | 1.25 (0.79-1.98) |
| 5 (most deprived) | 164 (10.8) | 45 (18.4) | 1.60 (0.98-2.61) |
| Unknown | 56 (3.7) | 5 (2.0) | 0.67 (0.25-1.81) |
| Comorbidities | | | |
| Cerebrovascular disease^c^ | 474 (31.3) | 77 (31.4) | 1.03 (0.76-1.41) |
| Ischaemic heart disease^c^ | 1070 (70.6) | 176 (71.8) | 1.01 (0.73-1.39) |
| Myocardial infarction^c^ | 410 (27.1) | 77 (31.4) | 1.24 (0.90-1.70) |
| Hypertension^c^ | 945 (62.4) | 144 (58.8) | 0.81 (0.60-1.09) |
| Hyperlipidaemia^c^ | 525 (34.7) | 92 (37.6) | 1.13 (0.84-1.53) |
| Diabetes mellitus^c^ | 253 (16.7) | 51 (20.8) | 1.19 (0.83-1.72) |
| Gout^c^ | 132 (8.7) | 17 (6.9) | 0.71 (0.41-1.25) |
| Rheumatoid arthritis^c^ | 45 (3.0) | 16 (6.5) | 1.26 (0.66-2.39) |
| Osteoarthritis^c^ | 654 (43.2) | 124 (50.6) | 1.15 (0.85-1.55) |
| COPD^c^ | 114 (7.5) | 29 (11.8) | 1.24 (0.77-1.99) |
| Asthma^c^ | 236 (15.6) | 41 (16.7) | 0.78 (0.52-1.15) |
| Anaemia^c^ | 125 (8.3) | 51 (20.8) | 2.47 (1.67-3.65) |
| Stress^c^ | 111 (7.3) | 30 (12.2) | 1.55 (0.98-2.46) |
| Anxiety^c^ | 261 (17.2) | 52 (21.2) | 1.13 (0.79-1.61) |
| Depression^c^ | 346 (22.8) | 80 (32.7) | 1.40 (1.02-1.94) |
| IBS^c^ | 90 (5.9) | 18 (7.3) | 1.05 (0.60-1.83) |
| GERD^c^ | 214 (14.1) | 48 (19.6) | 1.14 (0.78-1.67) |
| PUD-related symptoms^d^ | 317 (20.9) | 92 (37.5) | 1.80 (1.28-2.54) |

^a^OR adjusted for age, sex, follow-up time, health service utilization (PCP visits and referrals), smoking and drug use during the study period (gastroprotective drugs, NSAIDs, ASA and paracetamol).

^b^In year before the index date.

^c^Diagnosed before the index date. Relative to being free from the comorbidity.

^d^Diagnosed between the start date and the index date. Relative to being free from symptoms.

Abbreviations: *ASA*-Acetylsalicylic acid; *BMI*-Body mass index; *CI*-Confidence interval; *COPD*-Chronic obstructive pulmonary disease; *GERD*-Gastroesophageal reflux disease; *IBS*-Irritable bowel syndrome; *NA*-Not assessed; *NSAIDs*-Non-steroidal anti-inflammatory drugs; *OR*-Odds ratio; *PCP*-Primary care physician; *PUD*-Peptic ulcer disease.
