# Supplementary material for: Risk of uncomplicated peptic ulcer disease in a cohort of new users of low-dose acetylsalicylic acid for secondary prevention of cardiovascular events
Source: BMC Gastroenterol. 2014 Dec 10;14:205. doi: 10.1186/s12876-014-0205-y (PMC4272555; doi:10.1186/s12876-014-0205-y)
Supplement: Additional file 2: Table S2. — Odds ratios for the risk of uncomplicated peptic ulcer disease associated with current use of medications, nested case–control analysis restricted to current users of low-dose ASA at their index date. [file 12876_2014_205_MOESM2_ESM.doc]

Supplementary Table 2 Odds ratios for the risk of uncomplicated peptic ulcer disease associated with current use of medications, nested case–control analysis restricted to current users of low-dose ASA at their index date

|  | **Controls**  **N = 1515**  ***n* (%)** | **Uncomplicated PUD cases**  **N = 245**  ***n* (%)** | **Adjusted**  **OR (95% CI)^a^** |
| --- | --- | --- | --- |
| NSAIDs | 167 (11.0) | 50 (20.4) | 1.56 (1.06-2.30) |
| Selective COX-2 inhibitors | 24 (1.6) | 12 (4.9) | 2.53 (1.41-4.52) |
| tNSAIDs | 143 (9.4) | 38 (15.5) | 1.43 (0.93-2.17) |
| Paracetamol | 391 (25.8) | 95 (38.8) | 1.47 (1.05-2.05) |
| Clopidogrel | 93 (6.1) | 19 (7.8) | 0.98 (0.56-1.73) |
| Oral anticoagulants | 19 (1.3) | 2 (0.8) | 0.60 (0.13-2.71) |
| Dipyridamole | 54 (3.6) | 9 (3.7) | 0.87 (0.41-1.85) |
| PPIs | 361 (23.8) | 83 (33.9) | 1.61 (1.16-2.23) |
| Used on start date^b^ | 218 (14.4) | 37 (15.1) | 1.14 (0.75-1.73) |
| Use initiated after start date^c^ | 143 (9.4) | 46 (18.8) | 2.43 (1.60-3.69) |
| H_2_RAs | 43 (2.8) | 23 (9.4) | 2.56 (1.44-4.57) |
| Oral steroids | 39 (2.6) | 18 (7.3) | 2.14 (1.15-3.98) |
| SSRIs | 76 (5.0) | 22 (9.0) | 1.52 (0.90-2.58) |
| Tricyclic antidepressants | 105 (6.9) | 16 (6.5) | 1.14 (0.63-2.04) |
| Statins | 1168 (77.1) | 183 (74.7) | 1.03 (0.70-1.50) |

^a^OR adjusted for age, sex, follow-up time, health service utilization (PCP visits and referrals), smoking and drug use during study period (gastroprotective drugs, NSAIDs, ASA and paracetamol). Relative to non-use of drug.

^b^PPI therapy in use on the start date or started within the 30 days after the start date.

^c^PPI therapy started after the first 30 days of follow‑up.

Abbreviations: *ASA*-Acetylsalicylic acid; *CI*-Confidence interval; *COX-2*-Cyclooxygenase 2; *H_2_RAs*-Histamine-2 receptor antagonists; *NSAIDs*-Non-steroidal anti‑inflammatory drugs; *OR*-Odds ratio; *PCP*-Primary care physician; *PPIs*-Proton pump inhibitors; *PUD*-Peptic ulcer disease; *SSRIs*-Selective serotonin reuptake inhibitors; *tNSAIDs*-Traditional non-steroidal anti‑inflammatory drugs.
